# Mitotic Protein Kinase 1: Role in Spindle Assembly Checkpoint Revisited

**Published:** 2016-05-02

**Authors:** Anita Tandle, Kevin Camphausen

**Affiliations:** Radiation Oncology Branch, National Cancer Institute, National Institutes of Health, Bethesda, Maryland, USA

## Introduction

Cell division is a highly regulated process and involves sequential activation and deactivation of a number of proteins. Mitotic protein kinase (MPS1) is a part of the spindle assembly checkpoint (SAC) activated during prometaphase and metaphase that prevents chromosome misalignment by arresting the cell in mid-mitosis until all of the chromosomes is properly attached to the mitotic spindle [1]. Multiple cancers show alterations of the SAC machinery leading to chromosome missegregation and aneuploidy. MPS1 functions as a dual-specificity kinase that is critical for the recruitment of SAC proteins to unattached kinetochores, proper mitotic progression, and centrosome duplication [2]. Overexpression of Mps1 is observed in a number of cancer cell lines and tumor types, including breast cancer, gastric, colorectal cancer, anaplastic thyroid carcinoma, lung cancers and gliomas, also correlating with high histological grade and tumor aggressiveness [3,4]. High levels of Mps1 contribute to tumorigenesis by attenuating the spindle assembly checkpoint [5].

Inhibition of Mps1 activity can result in chromosome missegregation, aneuploidy, and eventually cell death. Thus, MPS1 may be an important target in cancer cells with dysregulated SAC and may be targeted via MPS1 inhibition. Recently several small molecule inhibitors of MPS1 have been developed and tested in pre-clincal models [6–10]. Two of them have entered human clinical trials (BAY 1161909, ClinicalTrials.gov Identifier: NCT02138812; BAY 1217389 ClinicalTrials.gov Identifier: NCT02366949). Majority of these inhibitors cause massive aneuploidy and cells ultimately succumbed to the mitotic catastrophe-induced activation of the mitochondrial pathway of apoptosis [7]. MPS1 inhibitors may exert robust anticancer activity, either as a monotherapy or in combination with other chemotherapy. In combination with an antimitotic cancer drugs it has not only enhanced their efficacy but potentially can overcame resistance to these agents [7,10].

Apart from being a major component of SAC machinery, MPS1 has been shown to be involved in turning on Smad signaling by phosphorylating Smad2/3 proteins [11]. Our own research has shown that MPS1 mediates phosphorylation of SMAD3 protein resulting in micro RNA-21 (miR-21) activation in GBM cells. Downregulation of MPS1 might upregulate the expression of the tumor suppressor PDCD4 and MSH2 genes, by down regulating miR-21 [12]. MPS1-induced Smad2 activation occurs in late G2 or early mitosis, indicating MPS1 mediated Smad activation is cell cycle-specific. This aligns well with the role of MPS1 being a mitotic protein kinase.

## MPS1 and Stress Responses

In addition to regulation of the spindle assembly checkpoint (SAC), MPS1 may also participate in other stress responses. It controls DNA damage responses and genome stability by phosphorylating the Bloom syndrome helicase (BLM), CHK2 in the G2/M checkpoint and c-ABL after oxidative stress [13–15]. MPS1-dependent BLM phosphorylation is important for ensuring accurate chromosome segregation [13]. Upon exposure to genotoxic stress, the c-Abl tyrosine kinase is released from cytoplasmic 14-3-3 proteins and targeted to the nucleus. Phosphorylation of Thr735 in c-Abl is critical for binding to 14-3-3. MPS1 is a physiological kinase that phosphorylates Thr735 and which is of importance to the cytoplasmic sequestration of c-Abl [15]. Previous studies demonstrated that MPS1, activated by DNA damage, phosphorylates CHK2 at Thr68, resulting in CHK2 activation and arrest of the cell cycle at G2/M. Reciprocally, MPS1 can be phosphorylated at Thr288 and stabilized by CHK2 after DNA damage, thus forming a positive regulatory loop [16]. The tumor suppressor protein p53 is another MPS1 substrate where phosphorylation by MPS1 disrupts p53-MDM2 interaction and causes stabilization and activation of p53 [17]. MPS1 phosphorylates the N-terminal domain of p53 at Thr18, and this phosphorylation disrupts the interaction with MDM2 and abrogates MDM2-mediated p53 ubiquitination. Phosphorylation at Thr18 enhances p53-dependent activation of not only p21 but also Lats2, two mediators of the post-mitotic checkpoint. These observations are important with respect to connecting the spindle checkpoint with p53 in the maintenance of genome stability [17]. Same group recently shown that, MPS1 also participates in the repair of oxidative DNA lesions and cell survival through the MDM2-histone2B axis [18]. In response to oxidative stress, MPS1 phosphorylates MDM2, which in turn promotes histone H2B ubiquitination and chromatin decompaction. These events facilitate oxidative DNA damage repair and ATR-CHK1, but not ATM-CHK2 signaling. Depletion of MPS1 or MDM2 compromised H2B ubiquitination, DNA repair and cell survival [18]. However, it is not known what triggers the selectivity of various MPS1 substrates either Smad-2, p53 or MDM2 and subsequent down stream effects

## DNA Damage Response

Eukaryotic cells are subject to tens of thousands of DNA lesions per day. In order to overcome the accumulation of DNA damage and to maintain genomic integrity, cells are equipped DNA damage response machinery to detect and repair these lesions. Recently we published studies examining effect of MPS1 inhibition on glioblastoma (GBM) cell growth *in vitro* and *in vivo* [19]. Inhibition of MPS1 activity resulted in reduced GBM cell proliferation by induction of mitotic catastrophe and abrogating its clonogenic potential. These effects were augmented when MPS1 inhibitor, NMS-P715 was combined with radiation. Repair of double strand breaks by ionizing radiation was compromised in the absence of MPS1. Furthermore, NMS-P715 in combination with fractionated doses of radiation significantly enhanced the tumor growth delay. Next, molecular profiling of MPS1-silenced GBM cells showed an altered expression of transcripts associated with DNA damage, repair, and replication, including the DNA-dependent protein kinase (PRKDC/DNAPK). The direct consequence of MPS1-mediated DNAPK downregulation was inhibition of two important DNA repair pathways; homologous recombination (HR) and non-homologous end joining (NHEJ). The reduced DNA repair efficiency in MPS1-inhibited GBM cells was associated with increased retention of gH2AX foci and induction of mitotic catastrophe. Thus, our model for sensitivity to MPS1 inhibition depends on NHEJ and HR deficiency in MPS1-depleted cells [19]. MPS1 inhibition can act as double edged sword; on one hand this will lead to an increase in genomic instability in cancer cells to levels that are incompatible with cancer cell survival and on the other inhibition of DNA repair will force cells through cell cycle to cause cell death. Inhibition of DNA repair holds great promise for damaging tumor cells. Thus, inhibition of DNA repair related function of MPS1 could represent a beneficial therapeutic strategy with enhanced therapeutic index. Therefore, this approach may be more widely applicable in the treatment of variety of cancers.

Although our research has shed light on the role of MPS1 in DNA damage responses, it raises an important question, “why would cells inhibit the recruitment of DNA repair factors during mitosis”? We know that NHEJ is active during mitosis; however does HR plays any direct role during mitosis? Clearly, additional research is required to further uncover the complex regulation of DNA repair during mitosis.

Personalized medicine using small molecule cancer therapeutics is the major focus of current biomedical research. Low molecular weight inhibitors of protein kinases represent a very attractive family of cancer drugs. Taken together, small molecule inhibitors of MPS1 could add important therapeutic benefit for the treatment of various cancers.

## References

[1] Lara-Gonzalez P, Westhorpe FG, Taylor SS (2012). The spindle assembly checkpoint. Curr Biol.

[2] Lauzé E, Stoelcker B, Luca FC, Weiss E, Schutz AR (1995). Yeast spindle pole body duplication gene MPS1 encodes an essential dual specificity protein kinase. EMBO J.

[3] Liu X, Winey M (2012). The MPS1 family of protein kinases. Annu Rev Biochem.

[4] Dominguez-Brauer C, Thu KL, Mason JM, Blaser H, Bray MR (2015). Targeting Mitosis in Cancer: Emerging Strategies. Mol Cell.

[5] Ling Y, Zhang X, Bai Y, Li P, Wei C (2014). Overexpression of Mps1 in colon cancer cells attenuates the spindle assembly checkpoint and increases aneuploidy. Biochem Biophys Res Commun.

[6] Colombo R, Caldarelli M, Mennecozzi M, Giorgini ML, Sola F (2010). Targeting the mitotic checkpoint for cancer therapy with NMS-P715, an inhibitor of MPS1 kinase. Cancer Res.

[7] Jemaà M, Galluzzi L, Kepp O, Senovilla L, Brands M (2013). Characterization of novel MPS1 inhibitors with preclinical anticancer activity. Cell Death Differ.

[8] Liu Y, Lang Y, Patel NK, Ng G, Laufer R (2015). The Discovery of Orally Bioavailable Tyrosine Threonine Kinase (TTK) Inhibitors: 3-(4-(heterocyclyl)phenyl)-1H-indazole-5-carboxamides as Anticancer Agents. J Med Chem.

[9] Naud S, Westwood IM, Faisal A, Sheldrake P, Bavetsias V (2013). Structure-based design of orally bioavailable 1H-pyrrolo[3,2-c]pyridine inhibitors of mitotic kinase monopolar spindle 1 (MPS1). J Med Chem.

[10] Wengner AM, Siemeister G, Koppitz M, Schulze V, Kosemund D (2016). Novel Mps1 Kinase Inhibitors with Potent Antitumor Activity. Mol Cancer Ther.

[11] Zhu S, Wang W, Clarke DC, Liu X (2007). Activation of Mps1 promotes transforming growth factor-beta-independent Smad signaling. J Biol Chem.

[12] Maachani UB, Tandle A, Shankavaram U, Kramp T, Camphausen K (2015). Modulation of miR-21 signaling by MPS1 in human glioblastoma. Oncotarget.

[13] Leng M, Chan DW, Luo H, Zhu C, Qin J (2006). MPS1-dependent mitotic BLM phosphorylation is important for chromosome stability. Proc Natl Acad Sci U S A.

[14] Wei JH, Chou YF, Ou YH, Yeh YH, Tyan SW (2005). TTK/hMps1 participates in the regulation of DNA damage checkpoint response by phosphorylating CHK2 on threonine 68. J Biol Chem.

[15] Nihira K, Taira N, Miki Y, Yoshida K (2008). TTK/Mps1 controls nuclear targeting of c-Abl by 14-3-3-coupled phosphorylation in response to oxidative stress. Oncogene.

[16] Yeh YH, Huang YF, Lin TY, Shieh SY (2009). The cell cycle checkpoint kinase CHK2 mediates DNA damage-induced stabilization of TTK/hMps1. Oncogene.

[17] Huang YF, Chang MD, Shieh SY (2009). TTK/hMps1 mediates the p53-dependent postmitotic checkpoint by phosphorylating p53 at Thr18. Mol Cell Biol.

[18] Yu ZC, Huang YF, Shieh SY (2016). Requirement for human Mps1/TTK in oxidative DNA damage repair and cell survival through MDM2 phosphorylation. Nucleic Acids Res.

[19] Maachani UB, Kramp T, Hanson R, Zhao S, Celiku O (2015). Targeting MPS1 Enhances Radiosensitization of Human Glioblastoma by Modulating DNA Repair Proteins. Mol Cancer Res.

